# Case Report—Extra-Uterine, Pregnancy, Four Years and Eight Months’ Duration, Complicated by Entero-Uterine Fistula

**Published:** 1890-12

**Authors:** R. R. Kime

**Affiliations:** Petersburg, Ind.


### CASE REPORT—EXTRA-UTERINE PREGNANCY—FOUR YEARS AND EIGHT MONTHS' DURATION—COMPLICATED BY ENTERO-UTERINE FISTULA.*

BY R. R. KIME, M. D., PETERSBURG, IND.

Mrs. B.—aged 27 at time pregnancy occurred; menstruated first at 17 years of age; never regular; always severe pains at flows, chills, at times delirious. Married at 22, first confinement 18 months afterward, severe labor, forceps used, child born dead, sick three months. Second confinement two years after first, not so severe, child born dead, placenta adherent.

Third and present pregnancy eighteen months after the second; first felt movements December 25th, 1885, continuing until night of January 18th, 1886, when she complained of severe pain and active movements after which she felt no further movements. Was called, arriving the morning of the 19th; found her resting comparatively well, no special evidence of severe shock, bowels somewhat tender, and enlarged by a tumor corresponding to a five months' pregnancy; no fœtal movements and no beat of fœtal heart. Gave treatment for what I supposed to be enteritis, complicated by malaria. Patient living seven miles in the country did not see her until 21st; and February 3d, not being satisfied with her condition, she believing herself pregnant, Dr C. H. Fullinwider was called in counsel February 5th, 1886, at which time she was having some hemorrhage from the bowels. After careful examination we decided the probabilities were that she was not pregnant. The fever and inflammatory symptoms gradually subsided. June 12th, 1886, four months afterward, she came to my office, and Drs. Adams and Fullinwider and myself examined her; found a tumor attached to and seemingly imbedded in right side and front wall of uterus, reaching one inch above umbilicus; measuring transversely one inch below umbilicus, was eight inches; longitudinally, it was five inches;

*Read before the Mississippi Valley Medical Association, Louisville, Ky., Oct. 10, 1890.

uterus five and one half inches in depth. From location of growth, firmness, attachments and history the case was diagnosed as fibroid tumor of the uterus, and treatment by electricity and ergot instituted, which resulted in marked diminution in size of tumor—at least one-fourth; with marked improvement in general condition she passed from under treatment during 1887 and 1888. Patient again called at my office February 11th, 1889, about two and one half years from last examination, complaining of increase in size of tumor, general health failing, at times suffering severe pains and having a bloody discharge. Used galvanic electricity, positive pole, in uterus, and prescribed ergot with tonics. Hemorrhage checked, leaving a bloody purulent discharge. Returned March 20th, saying tumor had dropped down, and complaining of weighty bearing down sensations in lower part of bowels. The tumor then measured seven and one-half inches, transversely four and one-half inches, and one and one-half inches lower in bowels, cervix more patulous and low down in vaginal canal. Continued ergot and tonics; applied electricity April 6th, 13th and 17th. She, returning home on the 17th did some stooping work; that night began to suffer severe pains, lasting through the next day, and next night they became so severe I was sent for; arriving next morning found patient resting quietly, a profuse discharge having set up in after part of night, which at the time I thought was due to the use of the electricity, but later proved to be rupture of fœtal cyst; the tumor became smaller and patient improved.

She again called at office March 19th, 1890, ten or eleven months from last examination, complaining of severe pains at times; emaciated, fever, with indications of septicæmia. It was then no difficulty on examinatiun to decide we had a case of extra-uterine pregnancy to deal with. By request she consulted Dr. S. E. Munford, of Princeton, Ind., who advised dilatation of cervix and removal of fœtal structures without abdominal section if possible. Commenced dilatation April 5th by tupelo and elm tents completed with Barnes bags after three days of very difficult and painful work. A canal three and one-half or four inches in length had to be dilated. On second day of dilatation corn husks and fecal material were found in uterine cavity, patient having eaten some corn four or five days previously.

Patient was anæsthetized April 8th, and the greater part of skull, spinal column, ribs and extremities of fœtus were removed.

Present Drs. S. E. Munford, A. R. Byers, C. H. Fullinwider and J. B. Duncan. After removal of the fœtal bones, the cavity was thoroughly irrigated and two large rubber drainage tubes introduced some eight or 9 inches into cavity, for drainage and irrigation. An enema next morning produced an evacuation of rectum, after which time the fecal discharges passed entirely through womb and vaginal canal for several days. A rectal injection of a pint or more of water would pass out through uterus and drainage tubes. Used both rectal and intra-uterine irrigations and liquid diet; kept tubes in three or four days; when they came out patient refused to have them reintroduced on account of pain.

At the end of twelve days the fecal discharges began to pass through their natural channel, and by the end of four weeks ceased passing by uterus entirely. Patient made a rapid recovery and is now enjoying good health, but from past experiences desires to avoid maternity, unless she can become a mother as other women do.

The above is the history of a case I shall now class as interstitial pregnancy. Of the later classifications of ectopic pregnancy, by authorities all forms are classed under three general heads, viz., ovarian, tubal, and abdominal, except Tait, who doubts the existence of the ovarian and excludes the primary abdominal. Tait's views as to the normal fecundation occurring in uterine cavity only and that all cases of erratic gestations are primarily tubal, which by primary and secondary rupture may become intra and extra-peritoneal, greatly simplify this vexed question. If it be true that fecundation occurs normally in uterine cavity only, we can better understand why ciliated movements in fallopian tubes are toward uterine cavity. Also better understand why disease of mucous membranes, lining fallopian tubes and dilatations of the same, favors ectopic pregnancy.

Zinke, in referring to this subject, states, "If Tait's views be correct, extra-uterine gestation would never occur for the following reasons :

"1st. Diseased tubes which would facilitate the passage of the germ imply a condition which renders the tube patulous throughout, in consequence of which the spermatozoa would obtain a ready entrance.

"2d. Tubes so affected would certainly as readily admit of the passage of an ovum, impregnated or otherwise, and thus rather tend to prevent than cause ectopic pregnancy."

The writer certainly failed to consider the migratory power of the spermatozoa to force its way out the patulous tube, even with less difficulty than in the normal state, for it would not have to oppose the ciliated motion. Also that the fecundation ovum has no inherent power of its own to force its way out the tube, the ciliated motion lost, rythmical action of tube nil, hence it would be inevitably left where fecundated.

We leave this question of normal fecundation occurring in uterine cavity only open for discussion.

To return to our case of interstitial pregnancy, which variety, Tait says,[1] "is rare and uniformly fatal by primary rupture befoıe the 5th month"—to briefly recapitulate, we have the history as follows : No flow from first conception, this being the third, no discharge of decidual membrane, no rupture unless on the night of the 18th of January, 1886, which condition did not indicate next morning, firmness of the tumor and situation, absence of fœtal movements or beat of fœtal heart; the case was diagnosed fibroid, but later, viewed from a different standpoint, we have reduction in size of tumor due to absorption of liquor amnii, two years later increase in size due to decomposition and pus formation, the bloody discharge the result of rupture of tissues between fœtal cyst and uterine cavity, dropping down of tumor was passage of fœtus into uterine cavity, the profuse discharge result of rupture of fœtal sac. The entero-uterine fistula was probably the result of penetration by fœtal bones of uterine or cyst wall and intestinal wall made adherent thereto by some

[1] Med. and Surg. Monographs, Vol. 5, No. 2, P. 469.

previous inflammation. The fistula gradually healed, patient improved rapidly and is now well and happy.

Pertaining to this subject we collect the following:

Tait says[2] "the interstitial variety cannot be diagnosed before rupture; that the rupture is intra-peritoneal."

E. E. Montgomery states,[3] "in the interstitial, form the rupture may occur either into uterine canal or abdominal cavity."

Maschka reports,[4] "a woman, twenty-nine years old, died suddenly, and criminal abortion being suspected, a judicial autopsy was made and an interstitial pregnancy with rupture was found; the body of the fœtus had passed into uterus and had been removed, but the head was torn from the body and remained in the sac."

Parks, of Chicago, reports[5] a case of interstitial pregnancy, with removal of products of conception through the uterine cavity.

Wathen, of Louisville, reports[6] a case of interstitial pregnancy rupturing into uterine cavity.

Hence, we conclude that it is almost mpossible to diagnose interstitial pregnancy before rupture, that rupture may occur into uterine canal as well as into abdominal cavity, and that it is not invariably fatal.

---

[2] Med. and Surg. Monographs, Vol. 5, No. 2, P. 518 and 469.
[3] Amer. Assoc. of Obst. and Gynecologists, Vol. 1, 1888.
[4] Wien. Med. Wochenscrift, 1885.
[5] Amer. Jour. of Obst., Vol. 20, P. 537.
[6] Amer. Jour. of Obst., Vol. 22, P. 791.

